# CompELS vs SELEX: Comparative Aptamer Discovery Integrating Rational Libraries and Enzymatic Elution

**DOI:** 10.1021/acsnanomed.6c00140

**Published:** 2026-09-02

**Authors:** Steven Ochoa, Valeria Tohver Milam

**Affiliations:** † School of Materials Science & Engineering, 1372 Georgia Institute of Technology, Atlanta, Georgia 30332, United States; ‡ Parker H. Petit Institute for Bioengineering and Biosciences, Georgia Institute of Technology, Atlanta, Georgia 30332, United States

**Keywords:** AlphaFold 3, competitive displacement, ligand, next generation sequencing, screening

## Abstract

Aptamers are oligonucleotides that fold into structures with target-binding activity comparable to antibodies, yet their discovery remains laborious and inconsistent. Traditional Systematic Evolution of Ligands by Exponential Enrichment (SELEX) screening often relies on randomized sequence libraries, repeated amplification, and harsh elution methods, each of which introduces bias and limits reproducibility. An alternative aptamer screening platform called Competition-Enhanced Ligand Selection (CompELS) employs repeated introduction of unenriched sequence populations while avoiding iterative elution and amplification steps. To elucidate key parameters governing selection outcomes, the effects of screening strategy, screening library design, and elution of bound DNA methodology were evaluated. A parallel study of candidate aptamer selection platforms against fluorescent protein mCherry revealed that SELEX required two additional enrichment cycles to achieve comparable enrichment of the same top 3 sequences selected via CompELS. A GT rich, empirically biased library was the predominant source of binding sequences, out-competing a tetraloop-structured library and random library. Finally, an enzymatic elution strategy enabled gentle recovery of aptamer-target complexes from either screening strategy to effectively reduce nonspecific carryover and improve the fidelity of each platform. These results demonstrate a streamlined, versatile aptamer candidate selection methodology that promises an accelerated aptamer discovery approach for protein targets and reduces bias by integrating CompELS, rational library design, and enzymatic elution.

## Graphical abstract

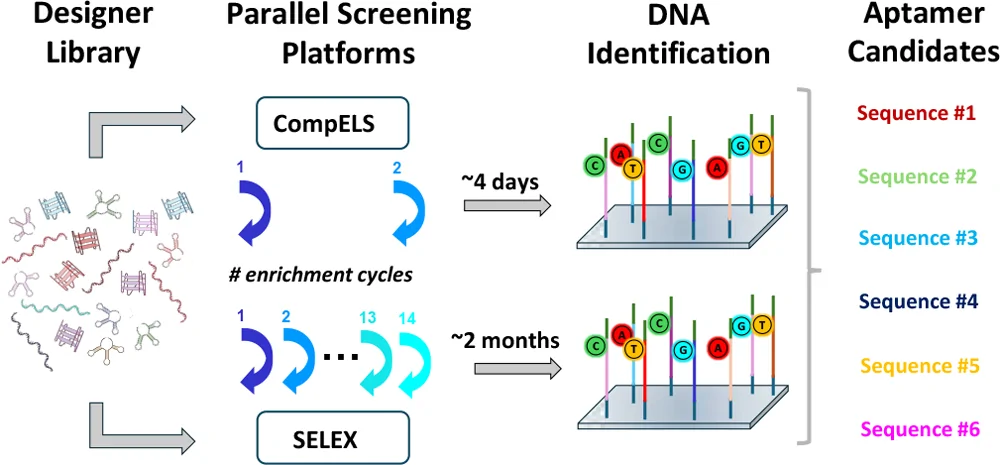

## Introduction

Aptamers are short, single-stranded oligonucleotides that fold into unique three-dimensional structures capable of binding to a wide range of targets with high specificity and affinity.^1–3^ Their advantages over traditional antibodies, such as in vitro chemical synthesis, low immunogenicity and ease of modification, position them as a promising class of affinity reagents and powerful tools in diagnostics, therapeutics and biotechnology.^4^ Their inherent versatility, including chemical or structural modification to reduce susceptibility to nuclease activity, promises broad capabilities ranging from acting as passive capture agents limited strictly to binding or tagging a particular target^5,6^ to active inhibitors of cellular activities.^7,8^ Historically, however, research and investment heavily favor engineered and humanized antibodies over aptamers with clinical trials of aptamers limited to select sequences, often structurally or chemically modified to enhance nuclease resistance.^9^ Despite the moderate success of the first FDA-approved aptamer, Macugen, which was later discontinued,^10^ the widespread clinical and commercial translation of aptamers has been further hindered by challenges in their discovery process.^11–13^ The conventional method for aptamer discovery called Systematic Evolution of Ligands by Exponential Enrichment (SELEX) has remained largely unchanged since it emerged three decades ago.^14–16^ SELEX is an iterative process that involves multiple successive rounds of incubating a vast, typically randomized oligonucleotide library with a target, partitioning, and finally amplification to enrich the next screening round library with previously bound sequences. While the SELEX methodology has been widely used, it is a slow and resource-intensive process with inherent bottlenecks stemming from oligonucleotide handling steps.^17–19^ Inherent bias from repeated PCR amplification steps can favor sequences that amplify efficiently rather than true target-binding sequences while harsh elution conditions, such as high temperature or extreme pH, can introduce carryover from nonspecific binding sequences and lead to false positives.^20–24^ Together, these SELEX-related challenges hinder progress toward more efficient screening, identification, and practical implementation of aptamers as diagnostic and therapeutic agents in nanomedicine.

Depending on the target size and chemical composition as well as on the partitioning method to separate unbound library from the target following incubation, the presentation of the target and oligonucleotide library during incubation can vary as follows: (1) free (i.e., nonimmobilized) target with a free oligonucleotide library;^25,26^ (2) free target with a free oligonucleotide library, followed by capture of the target-oligonucleotide complexes using bead substrates;^27^ (3) immobilized target with a free oligonucleotide library;^28^ or (4) free target with an immobilized library.^29–31^ New selection strategies to facilitate partitioning, fine-tune selection stringency, or reduce amplification-related errors have been developed to overcome the methodological limitations inherent to the SELEX procedure.^25,28,30,32–35^ One such approach, Competition-Enhanced Ligand Selection (CompELS), minimizes PCR cycles and harsh elution steps by continuously introducing fresh, unenriched libraries to incumbent aptamer-target complexes.^36–38^ Separate competitive displacement studies in oligonucleotide systems have successfully demonstrated achieving, for example, improved target-binding specificity in hybridization studies^39,40^ as well as dynamic reassembly^41–43^ and isothermal disassembly of DNA-linked colloidal particles.^44,45^ Motivated by these prior successes leveraging competitive exchange events, CompELS is designed to promote exchange or displacement of already bound sequences with potentially higher affinity candidates introduced each screening round to the desired target. Importantly, CompELS increases selection stringency by incorporating multiple rounds of competitive binding while preserving a high level of sequence diversity beyond the earliest screening rounds. Since the library is divided into several aliquots, CompELS also facilitates screening of large combinatorial libraries at appropriate concentrations without the need for large sample volumes. By limiting PCR to a single enrichment step at the end of each (of two) stages in the current study, PCR-induced artifacts can be inhibited and the CompELS workflow can be completed in a fraction of the time (approximately 2–4 days) with fewer reagents than conventional SELEX.

Methodological challenges are further compounded by the limitations of aptamer screening libraries used at the start of selection experiments.^46,47^ A typical aptamer screening experiment begins with approximately 10^15^ random oligonucleotides. While examples of well-studied aptamers with structures ranging from three-way junctions^48,49^ to G-quadruplexes^50–52^ have emerged from random sequence libraries, this population number represents only an infinitesimal fraction of the theoretical sequence space. Libraries with, for example, 40 nucleotide-long random segments yield 10^24^ possible sequence permutations. This undersampling means that optimal binders are likely to be absent from the initial pool.^53,54^ Moreover, random libraries are predisposed toward simple secondary structures such as hairpins (also known as stem-loops), a phenomenon known as the “tyranny of short motifs”.^53,55^ The resulting scarcity of structural diversity reduces the probability of discovering aptamers with complex tertiary architectures observed in naturally evolved functional biopolymers such as riboswitches and ribozymes.^56,57^ This conventional random sequence search approach to screening libraries makes aptamer discovery a relatively stochastic process, undermining its reproducibility and limiting its broader adoption into standardized biotechnological and clinical platforms. To improve the likelihood of identifying functional binders, rationally designed screening libraries characterized in a separate study^58^ have been explored in the current study to guide selection toward more promising domains of sequence space.^59–62^ These designer libraries incorporate compositional or structural motifs inspired by known functional nucleic acids such as biased nucleotide distributions or embedded structural elements.^63^

The present study systematically evaluates how screening strategy and library design influence aptamer discovery. The protein target, mCherry, is a red fluorescent protein with a characteristic β – barrel structure that is used as a reporter for *in vivo* imaging of tumor cells;^64^ monitoring the metabolic activity of gut microbial as candidate therapeutic agents;^65^ and to detect the presence of disease-causing pathogens.^66^ An equimolar mixture of three structurally distinct screening libraries includes a “Standard” conventional random library and two rationally designed libraries. The first designer library is an “Empirical” library containing GT rich sequences derived from compositional biases observed in known aptamers. The second designer library is a “Tetraloop” library incorporating thermodynamically stable tetraloop motifs to promote self-folding. The three sequence populations were mixed together and used in parallel aptamer selection experiments against a model target, fluorescent mCherry protein, to compare the efficacy of SELEX and CompELS. To further improve selection fidelity and minimize the risk of false positive identification, a gentle enzymatic elution strategy was introduced in which aptamer-target complexes are released from their immobilization matrix through enzymatic degradation of a Tobacco Etch Virus (TEV) cleavage site. This TEV elution approach was successfully used by Xiang et al. to recover membrane-bound protein targets, along with any bound DNA following Cell-SELEX.^67^ This approach enables the specific elution of aptamer-target complexes under gentle physiological conditions to reduce carryover of nonspecific binders. Overall, this study aims to elucidate events in the otherwise “black box”^46^ of aptamer selection by determining how screening library design and aptamer screening methodology shape aptamer selection.

## Results and Discussion

### Target-Specific Enzymatic Elution Improves Partitioning Fidelity

Details of the three templates used in the screening library, their handling during screening via exposure to negative target then to positive target, and postenrichment purification (Figure S1 in Supporting Information) are provided in the Methods section. Similarly, biotinylated mCherry target synthesis confirmed via PAGE (Figure S2 in Supporting Information) and streptavidin–biotin coupling to a resin substrate are detailed in Methods section. While target immobilization on a substrate facilitates separation of unbound library members, significant nonspecific binding of sequences during aptamer selection can occur in the presence of a solid phase support matrix.^68–70^ This persistent challenge in aptamer discovery can obscure the enrichment of true target binders and lead to poor partitioning efficiency and false positives. To overcome this challenge, a partitioning system was developed to reduce nonspecific library retention by optimizing washes and by incorporating a gentle elution method involving a cleavable linker between the mCherry protein target and the NeutrAvidin-coated resin.

First, to minimize nonspecific adsorption of the DNA library to the NeutrAvidin Ultralink resin as well as the polyethylene column used in this study, the effect of BSA addition to the selection buffer was evaluated. Here, DNA was incubated with the resin-packed column. Columns were then washed 15 times and the amount of DNA eluted was measured via qPCR before (wash 0) and after wash steps 1, 5, 10, and 15. As shown in Figure 1A, nanogram levels of DNA were measured by qPCR analysis of wash supernatants even after 15 consecutive washes with BSA-free blocking buffer. In contrast, by preblocking the beads and column with selection buffer containing 1% w/v BSA, more DNA was measured in the earliest wash, but significantly less DNA was recovered from the BSA-blocked column compared to the BSA-free column by wash 5 onward with only picogram levels by wash 15. This reduction in DNA measured in the flow through at later wash steps is likely due to fewer nonspecific binding surfaces available in the presence of BSA blocking agents. Thus, initial studies revealed that preblocking with 1% w/v BSA successfully mitigated nonspecific DNA binding to the polyethylene column and beaded polyacrylamide resin.

**Figure 1:**
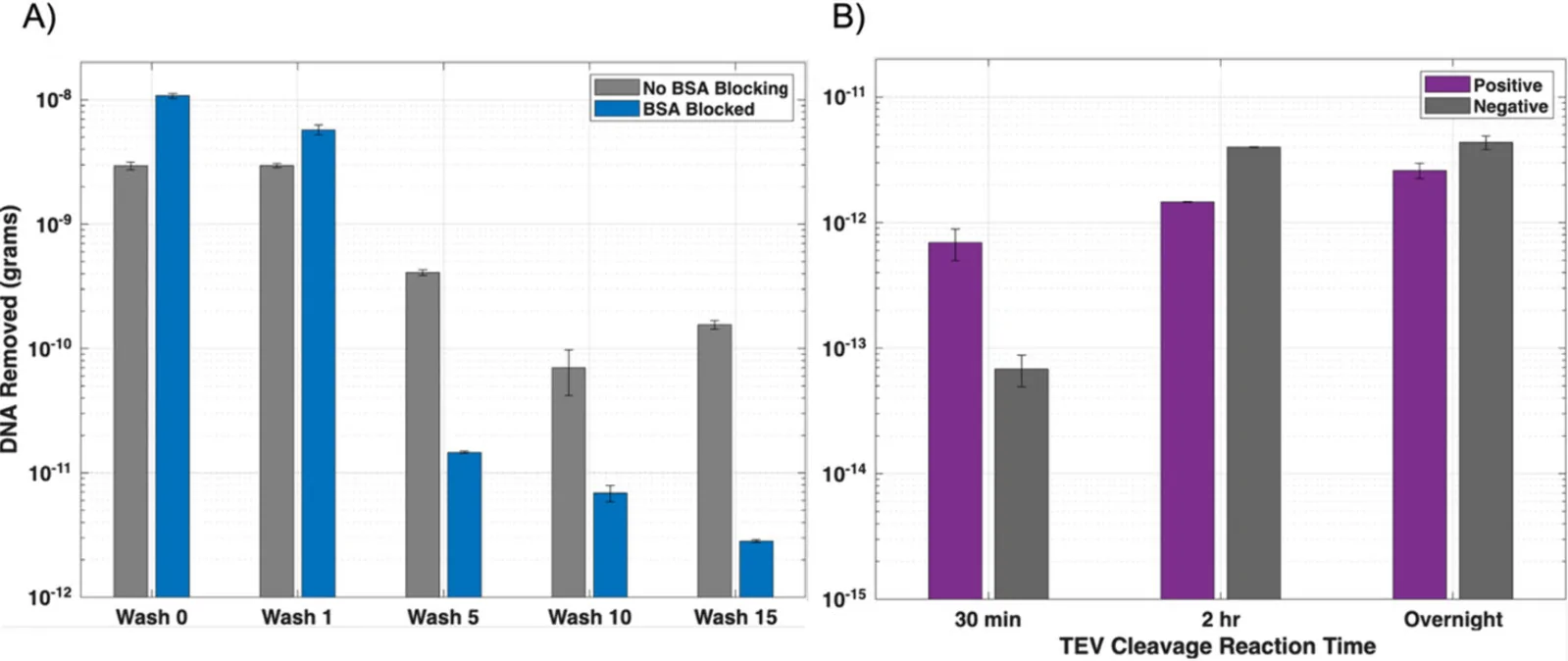
Optimization of the partitioning system to minimize nonspecific library retention. (A) qPCR quantification of nonspecific DNA removed from bare (i.e., no mCherry) NeutrAvidin resin-packed column after sequential washes to compare the effects of no BSA blocking (gray) with 1 w/v% BSA blocking (blue). (B) Elution of DNA following incubation with TEV protease cleavage of mCherry-coupled resin, preblocked with BSA (positive sample, purple) and bare resin preblocked with BSA (negative sample, dark gray). Error bars represent standard deviation of three samples.

To further reduce binding prior to aptamer screening, a counter-selection step was performed by incubating the naive library with a target-free, BSA-blocked resin-packed column then collecting unadsorbed library members in the flow-through for subsequent screening against the positive target. Furthermore, after library incubation with immobilized mCherry during screening, the column was subjected to multiple stringent washes with a high-ionic strength buffer (selection buffer supplemented with 2 M NaCl, pH 8.5) to remove as many nonspecific or weakly bound sequences as possible. The collective data in Figure 1A indicates that this combination of BSA blocking and stringent washing appeared to reduce nonspecific library retention to picogram amounts. Since these steps preceded the buffer-induced re-equilibration of target-immobilized resin with any remaining DNA binders, this approach provided a suitable protocol for subsequent screening studies with resin-immobilized mCherry.

Second, a gentle elution method was employed to specifically isolate aptamer-target complexes and to circumvent complications encountered when using typical elution methods such as harsh chemical or thermal denaturation which can coelute nonspecific binders. The mCherry target was immobilized via a linker containing a TEV protease cleavage site and is designed to specifically release only aptamer-target complexes under mild physiological conditions.^71^ Importantly, the bound mCherry protein does not interfere with subsequent processing and sequencing steps.

Separate optimization experiments were performed to maximize the signal-to-noise ratio of DNA recovered following TEV protease cleavage of mCherry protein. Positive (mCherry-coupled resin) and negative (bare resin) controls were incubated with naive library with each resin condition subjected to the optimized blocking and stringent wash protocol discussed above and then treated with a TEV protease cleavage reaction for incubation times of 30 min, 2 h or overnight. In contrast to the two longer incubation times, the 30 min cleavage reaction using 20 units of TEV protease produced ∼10-fold greater recovery of DNA from the positive column compared to the negative control (Figure 1B). Thus, this 30 min cleavage window maximized the specific release of target-bound aptamer complexes before the signal-to-noise ratio was diminished by the slower, likely passive dissociation of DNA bound through nonspecific interactions. These optimization studies confirmed that this combined strategy of blocking, stringent washing and gentle enzymatic elution work together as a high-fidelity partitioning system.

### CompELS Promoted Accelerated Selection Compared to SELEX

With a high-fidelity partitioning method to minimize nonspecific library retention established, a 14-cycle SELEX experiment was performed in parallel with a two-stage CompELS protocol using an equimolar mixture of the three libraries split into two starting pools for each platform as illustrated in Scheme 1. Each SELEX enrichment cycle consisted of library-target incubation followed by aptamer-target complex elution and then PCR amplification of the bound library. In contrast, each stage of CompELS consisted of 10 rounds of target incubation with separate, unenriched screening library aliquots and no intermittent PCR amplification steps. Only at the end of each CompELS stage were the isolated target-bound sequences PCR amplified.

**Scheme 1:**
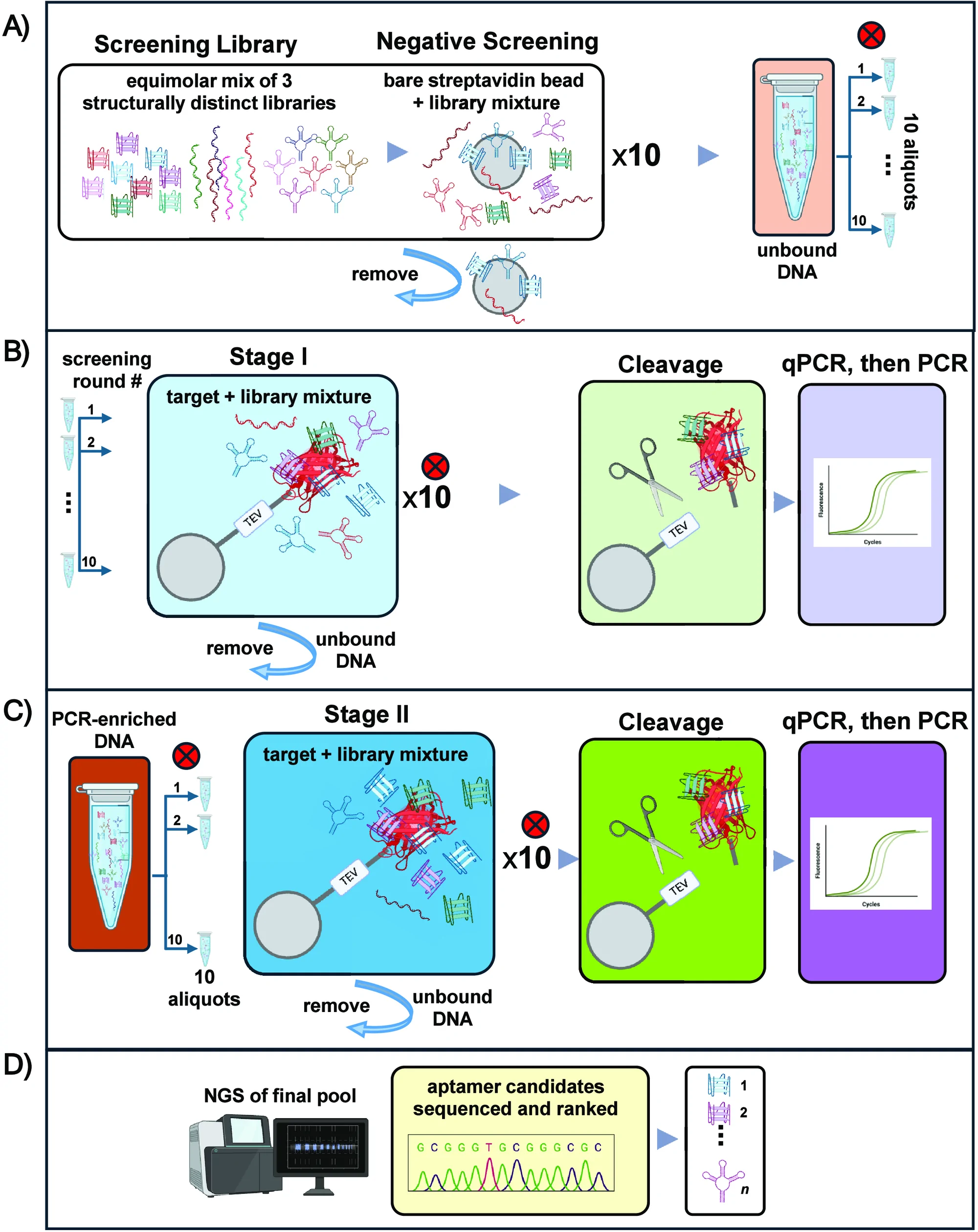
Schematic Illustration Highlighting Key Steps in CompELS Screening^a^

a Starts with (A) negative screening against streptavidin substrate to remove nonspecific binders from the library; nonbinding DNA is pooled and then split into 10 aliquots. (B) During Stage I, each library aliquot is then incubated with the immobilized target followed by stringent washes to remove unbound DNA; following 10 rounds of target incubation + wash, the target-DNA complex is cleaved, evaluated via qPCR, and then PCR enriched. (C) PCR-enriched DNA library is then split into 10 aliquots to undergo Stage II followed by qPCR and then PCR enrichment. (D) Final DNA aptamer candidate pool is prepared for NGS as detailed in Ochoa and Milam,^58^ sequenced and ranked according to frequency among identified sequences. Key steps excluded from SELEX screening are marked with a red, circled X.

The convergence of the SELEX library diversity as a function of enrichment cycle was monitored in real-time using qPCR melt-curve analysis (Figure 2). In this assay, the library melt temperature(s) depend on the relative amounts of imperfectly matched heteroduplexes expected for a highly diverse sequence population and perfectly matched oligonucleotide homoduplexes expected for a sequence population of low diversity. As shown in Figure 2, candidate pools at the start of screening collectively melt at a lower temperature (∼68 °C). As screening progresses during a series of 14 enrichment cycles, this lower melting temperature peak area continuously decreases while a higher melting temperature peak of ∼83 °C emerges. This gradual shift in melting temperature values of a sequence population as a function of enrichment cycle is characteristic of a converging screening library and serves as a benchmark for the end of SELEX.^72–74^ Thus, the melt-curve analysis reflects the screening cycle-dependent progression of SELEX with the SELEX candidate pool remaining highly diverse for the first eight rounds of selection. The gradual transition to a high melting temperature peak, signaling enrichment, only begins to emerge at round 8 and requires 14 full SELEX screening rounds to converge to a single peak centered around 83 °C. This evolution profile is typical of conventional SELEX and represents the slow iterative timeline of screening.

**Figure 2:**
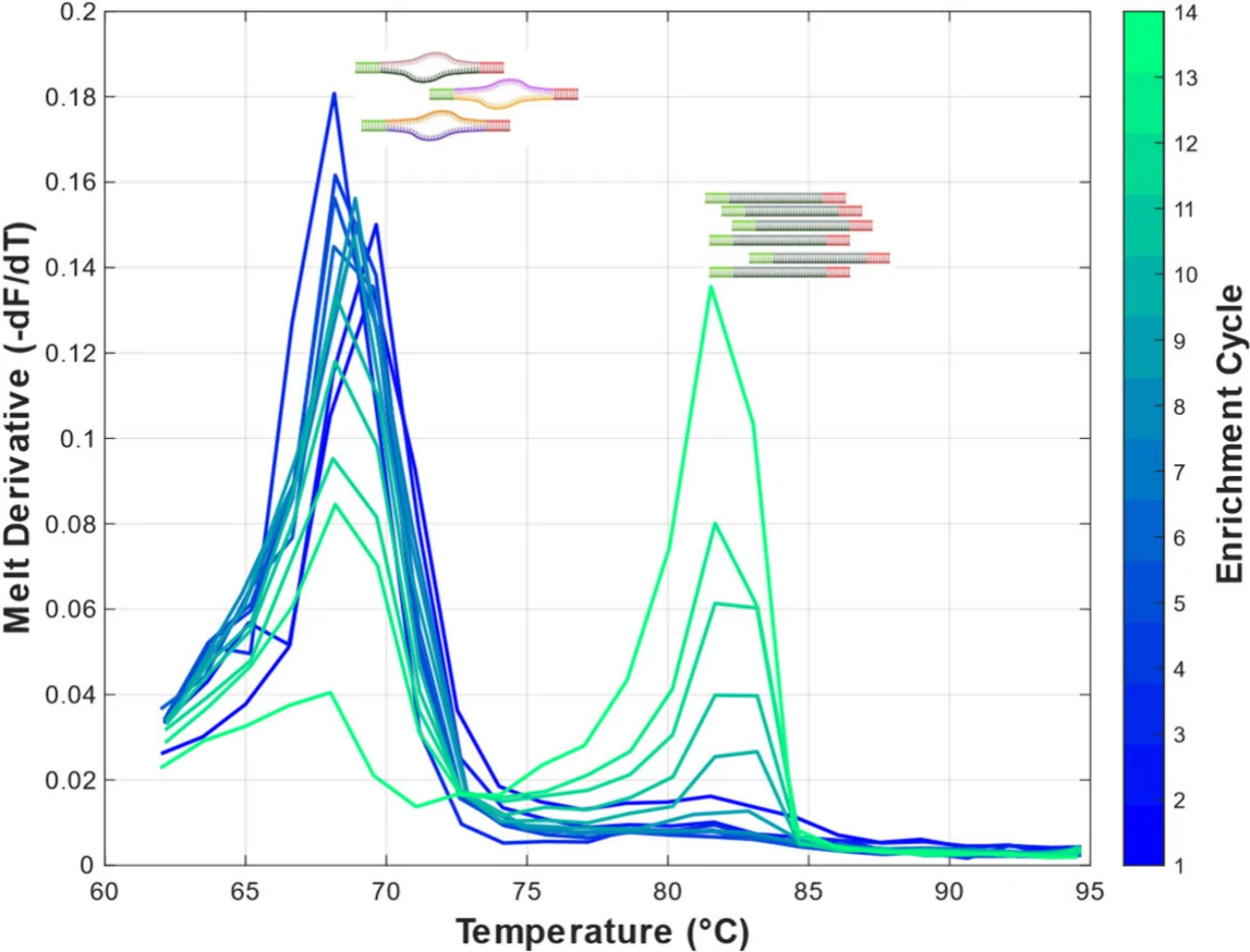
Monitoring of SELEX candidate pool convergence. qPCR analysis of melt-curve derivatives of the SELEX pools from enrichment cycle 1 (blue) through cycle 14 (green) with accompanying schematics of lower *T*_m_ heteroduplexes and higher *T*_m_ homoduplexes.

NGS analysis of sequence population evolution provides a more detailed quantitative comparison of selection efficiencies between SELEX and CompELS (Figure 3). Since the CompELS protocol is designed to minimize enrichment cycles by limiting PCR to occur only after each of two stages, a direct comparison of the sequence multiplicity after the first two enrichment cycles in both protocols is most revealing. As shown in the inset in Figure 3A after two enrichment cycles, the SELEX pool was still overwhelmingly unique as only 3.16% enriched of the library contained sequences with 2 or more copies. This enriched fraction was composed almost entirely of duplicate sequences (3.07%) and triplicate sequences (0.08%) with small subpopulations of the most abundant sequences present having only 3–4 copies (0.003%). In contrast, the CompELS pool was already 8.3% enriched by its second enrichment cycle. Critically, dominant populations with copy numbers exceeding 10, 100 and even 1000 copies emerged by the first enrichment cycle with the single most abundant family in the CompELS pool already accounting for 1.44% of all reads. This divergence trend demonstrates that the selection pressure in CompELS drives library convergence toward aptamer candidates with fewer enrichment cycles than SELEX.

**Figure 3:**
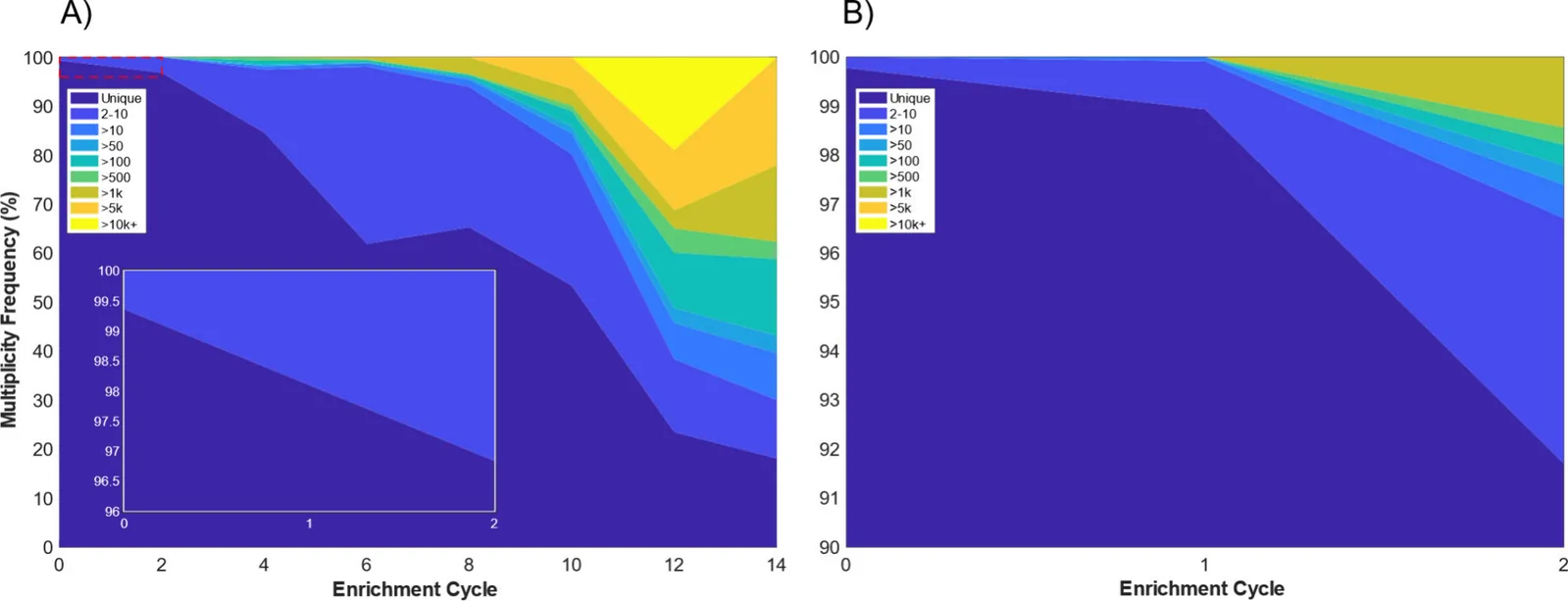
Comparative NGS analysis of sequence distribution within library population during each enrichment cycle of two screening platforms. Sequence multiplicity plots as a function of enrichment cycle number for (A) SELEX and (B) CompELS. Sequence copy number is indicated in the legends. Inset in (A) shows a zoomed in view of the plot region (enrichment cycles 0–2) outlined by the dashed red box in the top left corner.

### Comparative Library Enrichment Dynamics Reveals Advantage of CompELS

Further NGS analysis of sequence populations revealed that both SELEX and CompELS converged on several of the same sequence clusters with six of the most dominant sequence clusters appearing in both final pools (Table 1). While these sequence families appear as highly enriched clusters in the final pools from both selections, they were not detected in the naive pools, as expected for such undersampled libraries. However, the expected number of identical sequences within the library mixture of 10^15^ members with 10^24^ combinatorial possibilities is ∼4 × 10^5^. That number grows substantially if one includes closely related sequences. Based on this probability calculation anecdotally known as the “birthday paradox”, it is likely that the winning sequences were available to both libraries, illustrating that CompELS appears to be more efficient at enriching winners than SELEX. Alternatively, we cannot rule out low-level contamination from binding sequences isolated during optimization of the partitioning strategy, but the efficient nature of the CompELS technique is still evident.

**Table 1:** Top Six Aptamer Candidate Sequences (Seq1–Seq6) and One Low-Frequency Sequence (Seq7) Identified Following the Completion of SELEX and CompELS Screening^a^

		SELEX			CompELS		
Seed Sequence ID	Library	Cluster Rank in Final Pool	# Unique Sequences in Cluster	Avg. LED from Seed Sequence	Cluster Rank in Final Pool	# Unique Sequences in Cluster	Avg. LED from Seed Sequence
Seq1	Empirical	1	88	1.3	2	23	1.0
Seq2	Standard	2	82	1.1	3	18	1.0
Seq3	Empirical	3	145	2.0	1	65	1.9
Seq4	Standard	4	42	1.4	5	2	0.5
Seq5	Empirical	5	23	1.3	n/a	0	n/a
Seq6	Empirical	6	5	1.0	6	2	0.5
Seq7	Tetraloop	n/a	1	n/a	n/a	0	0.0

a For each aptamer candidate or “seed sequence” identified via NGS the library of origin, final rank by abundance, number of unique sequence mutations within each cluster family, and the average Levenshtein edit distance (LED) from the seed sequence of each cluster are listed.

Figure 4 tracks the emergence of these top six or the most dominant sequences identified in the final pools as a function of enrichment cycle for both CompELS and SELEX. The inset for Figure 4 illustrates early stage nuances for the top three sequence clusters. For example, the enrichment profile of one top candidate, the seed sequence for Cluster 3 (hereafter referred to as Seq3), clearly illustrates the performance disparity. In the CompELS workflow, Seq3 was immediately detectable after the first enrichment cycle appearing as 85.8 reads per million (RPM) in the pool and, after the second enrichment cycle of CompELS, became the most dominant sequence by enriching to 5625.0 RPM within the final CompELS pool. In contrast, this exact same sequence was not detected in the SELEX pool after the second cycle of enrichment. It required four enrichment cycles of SELEX for Seq3 to emerge at a comparable frequency (5879.2 RPM). By the 14th enrichment cycle, this same cluster had shifted from the most prevalent to the third most prevalent cluster in SELEX. More broadly, by the second enrichment cycle of SELEX, none of the top three sequence candidates were detected yet. In contrast, by the second enrichment cycle of CompELS, the top 3 clusters, namely, Seq3, Seq2, and Seq1 are identified, collectively accounting for 1.39% of the total pool.

**Figure 4:**
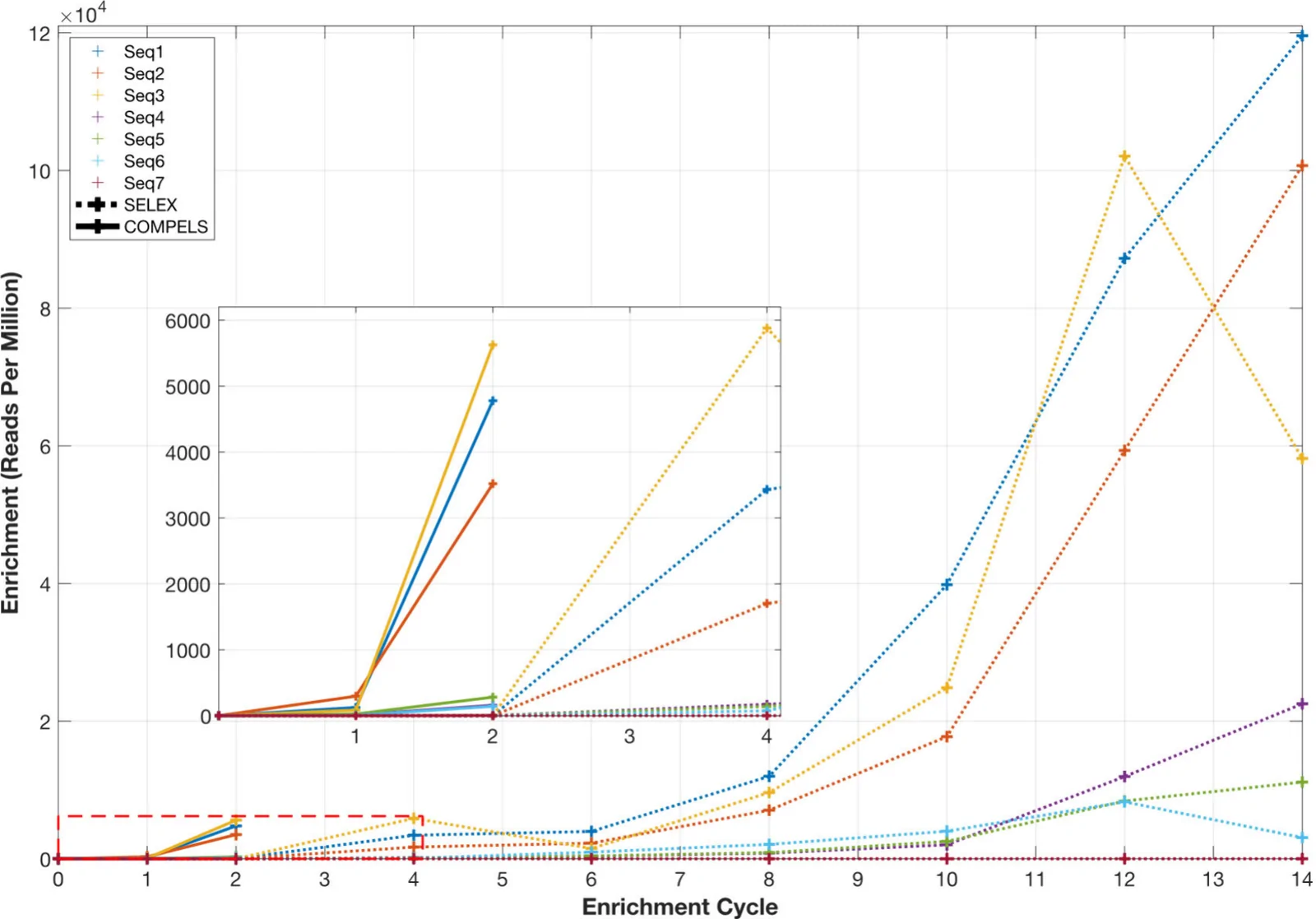
Enrichment dynamics of the top six sequences. Comparison of sequence enrichment (Reads Per Million, RPM) over 14 SELEX enrichment cycles (dotted lines) and two enrichment cycles following completion of each of two CompELS stages (solid lines). The inset magnifies critical early cycles (0–4) to pinpoint aptamer emergence for each selection platform.

A detailed comparison of enrichment dynamics between cycles (Table 2) further quantifies this difference in enrichment efficiency. As Table 2 shows, CompELS achieved significantly greater cycle-to-cycle fold-enrichment values for all six of the top candidate sequences in a *single* enrichment cycle than any *two* SELEX enrichment cycles. CompELS ultimately enriched the top candidate (Seq3) by ∼66-fold in a single enrichment cycle between cycle 1 when Seq3 first appears to the second and final enrichment cycle in the CompELS protocol. The SELEX workflow, however, required ten full enrichment cycles between cycle 4 when Seq3 first appears to the final SELEX cycle 14 to achieve a total enrichment of only ∼9.9-fold for the same seed sequence. This result provides a clear measure of selection efficiency demonstrating CompELS was more effective at enriching the top aptamer candidates with a total of only two enrichment cycles.

**Table 2:** Fold-Enrichment Analysis for the Top Six Candidate Sequences Where LED Is Zero^a^

		Fold Enrichment					
LED = 0	Cycle	Seq1	Seq2	Seq3	Seq4	Seq5	Seq6
SELEX	4 to 6	1.16	1.34	0.26	2.12	2.92	13.00
	6 to 8	3.01	3.10	6.40	2.23	2.19	2.17
	8 to 10	3.32	2.51	2.58	2.48	2.82	1.91
	10 to 12	2.19	3.34	4.11	5.86	3.31	2.06
	12 to 14	1.37	1.70	0.57	1.88	1.33	0.37
**SELEX (total)**	**4 to 14**	**34.85**	**59.12**	**9.89**	**129.69**	**79.31**	**41.46**
**CompELS**	**1 to 2**	**38.11**	**11.85**	**65.57**	**12.06**	**10.69**	**21.93**

a Comparison of fold-enrichment values between key cycles for CompELS and SELEX. The bolded bottom rows show the total fold-enrichment from the cycle of first appearance to the final cycle.

Notably, the extensive PCR amplification across multiple enrichment cycles during SELEX introduced significant mutational drift of sequence clusters as shown in Table 1. By cycle 14, the original Seq3 had spawned 145 closely related sequence populations bearing an average Levenshtein edit distance (LED) of 2 mutations. Including these mutated sequence families and generating sequence clusters using an LED = 10 reveals that Cluster 3 is also actually the most populous family in the pool (Table 1) despite Cluster 1 appearing as the numerically most enriched single-sequence family in the final SELEX pool (Figure S3 in Supporting Information). CompELS, by contrast, rapidly isolated the parent sequence with less sequence divergence among the top six sequences (Table 1).

### Empirically Biased Library Design Outperforms Random Sequence and Tetraloop Libraries

This study also provided a direct test of the performance of rationally designed screening libraries by including a mixture of a Standard (random) library, an Empirical (GT-rich) library and a Tetraloop (prestructured) library in the initial naive screening pool. The distinct thermodynamic and compositional properties of these libraries were previously characterized and validated, confirming they have unique structural characteristics and explore separate regions of primary and secondary structure space.^58^ Moreover, the identity of each of these libraries was encoded within a 5 nucleotide barcode sequence downstream of the fixed base segment at the 5′ end to enable analysis of comparative library performance every other enrichment cycle during SELEX.

As shown in Figure 5, however, while the mixture of all three barcoded libraries was prepared in equimolar concentrations, NGS analysis of the naive (Cycle 0) pool revealed differences in the identities of starting library members composed of ∼50% Tetraloop, ∼35% Standard and only ∼15% Empirical library sequences for both CompELS and SELEX. Since the libraries were carefully prepared to achieve this equimolar mix, this skew may reflect known difficulties in sequencing G-rich libraries on the Illumina platform.^75,76^ Prior studies indicate that these difficulties may be attributed to polymerase stalling on these G-rich segments, possibly due to overly stable G quadruplexes or even poor base calling during sequencing caused by low nucleotide complexity libraries on Illumina platforms.^77–79^ Despite this initial bias against identifying Empirical library members, sequences from the Empirical library comprehensively outcompeted both the Tetraloop and Standard libraries during aptamer screening with both selection platforms as shown in Table 1. After the eighth cycle of SELEX, the enrichment of the Empirical library became pronounced enlarging to nearly 50% of the final enriched pool by round 14. In fact, four of the top six (Seq1, Seq3, Seq5 and Seq6) sequence candidates originated from the Empirical library (Table 1). The Standard library was initially enriched in early cycles of SELEX, peaking at 65% of the pool by screening cycle 6, but eventually declined to ∼40% of the final pool (Cycle 14). Most notably and despite any favorable bias in identifying naive Tetraloop library members, this library appeared to perform the worst by immediately undergoing negative enrichment during SELEX and continuously being outcompeted until it accounted for just 2% of the final pool with none of its members among the top 6 sequence candidates or families shown in Table 1. This same trend in the various libraries did not occur during CompELS; however, this difference may be due to using only two enrichment cycles. Compared to SELEX, this reduced sequence divergence during CompELS likely reflects both its fewer PCR enrichment steps and its accelerated enrichment of top candidates. While evolutionary trends for the three screening libraries are evident during the 14 enrichment cycles for SELEX, further comparison of library trends between CompELS and SELEX is not possible due to the large numerical difference in enrichment steps for the two screening platforms.

**Figure 5:**
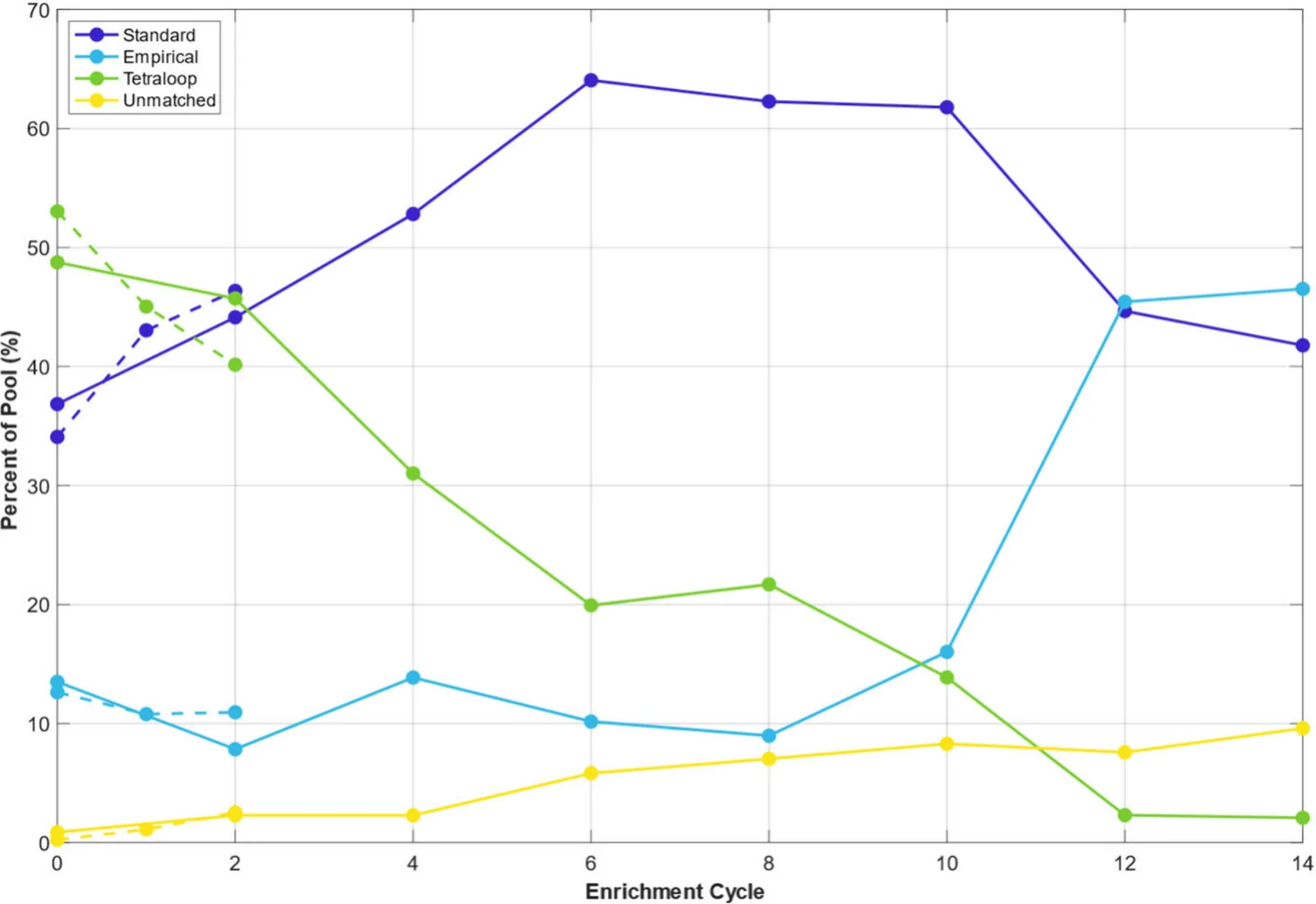
Comparative enrichment dynamics of structurally distinct screening libraries within aptamer screening pools as a function of enrichment cycle during CompELS (dashed lines) and SELEX (solid lines). The percentage of sequences originating from the Standard (dark blue), Empirical (light blue), Tetraloop (green), and Unmatched (yellow) within the total pool determined by NGS analysis of barcode identities.

The success of the GT-rich Empirical library, whose design was guided by empirical nucleotide composition trends observed in analysis of known aptamer sequences in the PDB, suggests that biasing aptamer screening libraries toward compositional space where validated aptamers have been empirically derived is a highly effective strategy. The poor performance of the Tetraloop library, designed with thermodynamically stable tetraloop hairpins between the two fixed base segments, implies that such a design may have imposed too much of a compositional constraint for this particular protein target. The prestructured tetraloop motifs may have resulted in highly stable structures that are incompatible with PCR amplification or simply may have restricted the scope of secondary structures accessible in the Tetraloop library and limited the conformational flexibility necessary for binding to this specific target.

### Aptamer Characterization and Validation

The seed sequences from the top six enriched clusters were synthesized for further characterization. An additional sequence, Seq7, which originated from the Tetraloop library and was present as only a single copy in the final SELEX pool and undetected in the final CompELS pool, was included as a low-enrichment representative. Binding affinity to the mCherry target was quantified using electrophoretic mobility shift assays (EMSA), as shown in Figure 6.

**Figure 6:**
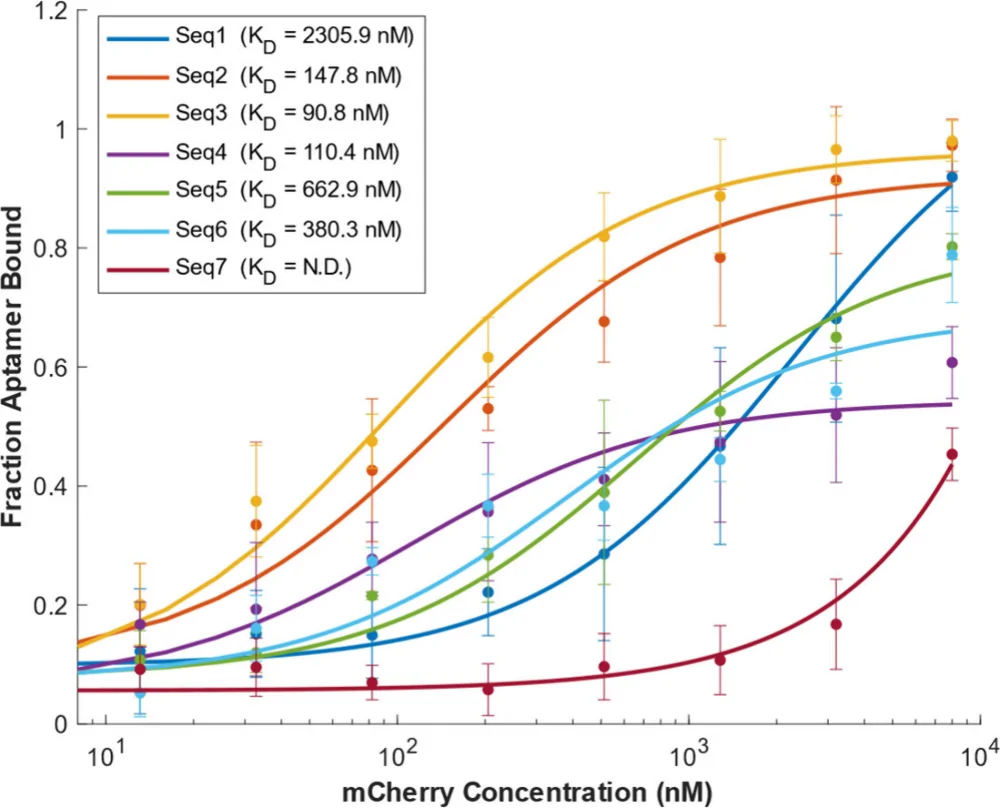
Binding affinity of selected aptamer candidates for mCherry. Electrophoretic mobility shift assay (EMSA) binding curves for the top six enriched sequences (Seq1–Seq6) and one low-enrichment representative (Seq7) against mCherry. Error bars represent standard deviation of three samples.

All six of the top candidates demonstrated distinct concentration-dependent binding interaction with mCherry, correlating with the NGS data. Seq3, the most dominant candidate in both selections, exhibited the highest binding affinity with an equilibrium dissociation constant, K_D_, of 90.8 nM while showing no detectable binding interaction with a negative control protein (streptavidin) (Figure S4B in Supporting Information). Notably, streptavidin-coated resin beads serve as the negative target during the prerequisite negative selection prior to mCherry exposure for both CompELS and SELEX screening platforms. The second most abundant candidate, Seq2, was also highly enriched in the final selection pools and exhibited a K_D_ of 147.8 nM. In contrast, the low-enrichment Seq7 showed minimal interaction and did not reach binding saturation.

Compositional analysis of the nucleotide distributions among the candidate sequences (Table 3) revealed that all six dominant sequences were guanine rich. This G-enrichment was not limited to the Empirical library as two of the sequences originating from the Standard library also exhibited high guanine content. Notably, in four of the top six sequences (Seq1, Seq3, Seq4 and Seq5) thymine was the second highest nucleotide percentage reflecting the Empirical library’s compositional design. The fact that the GT-rich (53.3% G, 24% T) binder Seq4 and the highly G-rich (68.9% G) Seq2 were both the only two sequences resulting from the Standard library among the top 6 candidates demonstrates that the compositional sequence space represented by the Empirical library is conducive for identifying functional aptamer sequences for the chosen mCherry target. In contrast the worst performing aptamer candidate, Seq7, had a relatively equivalent composition across all four nucleotides. Thus, despite any factors hindering NGS identification of Empirical library members in the naive library (cycle 0 in Figure 5), the preponderance of G-rich sequences among the top aptamer candidates in the current work appears to illustrate preferential selection of G-rich sequences during multiround aptamer screening. G-rich aptamers have been reported in literature reviews^80,81^ and individual reports of aptamers selected from random libraries.^50,82–85^

**Table 3:**
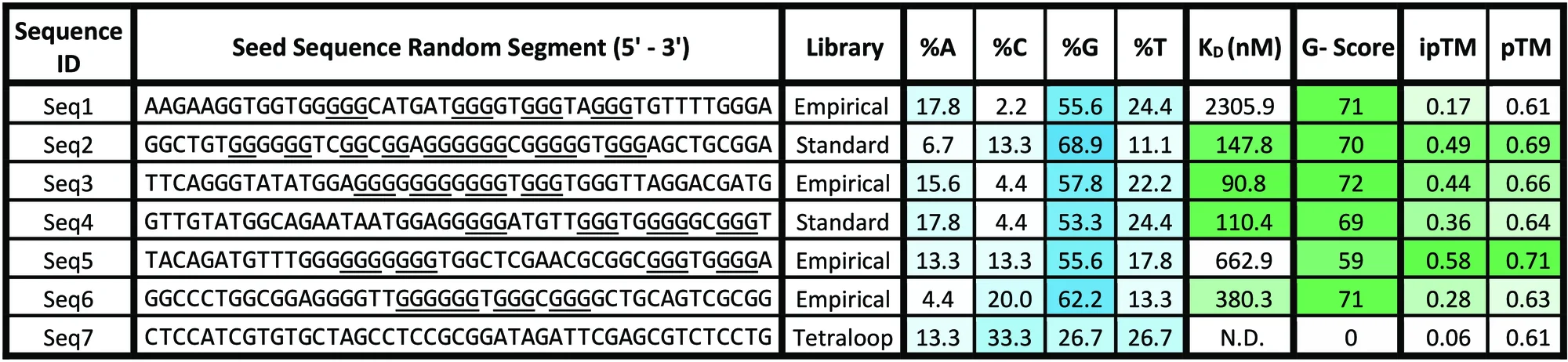
Central Segment of Sequences Selected for Further Characterization with Their Sequence Identity, Library of Origin, Respective Nucleotide Compositions, Equilibrium Dissociation Constant (K_D_), G-Score Value from G-Quadruplex Prediction by QGRS Mapper (with guanine residues predicted to participate in quadruplex formation underlined), Confidence Metrics of Interfacial Predicted Template Modeling (ipTM), and pTM for AlphaFold 3 Predictions of Complex Formation with mCherry with Color-Coded Gradients to Rank Values within Each Category

The ability of G-rich sequences to form three-dimensional G-quadruplex structures and noncanonical nucleobase interactions is well documented, a structural feature common among well-studied aptamer sequences.^86,87^ Given the observed high guanine content, sequences were analyzed for G-quadruplex potential using QGRS mapper.^88,89^ All six top candidate sequences, especially Seq3, exhibited strong G-scores (Table 3), suggesting these sequences are favored to form G-quadruplex structures though actual G-quadruplex structures are not generated via QGRS. Predicting the self-folded structures of these sequences with mFold, on the other hand, yielded no G quadruplex structures since this traditional secondary structure prediction tool only considers structures stabilized by canonical Watson–Crick base pair interactions. Recently, AlphaFold 3 has emerged as a promising computational tool capable of accurately predicting 3D aptamer structures, including G-quadruplex topologies, as well as 3D structures of aptamer-target complexes included in the PDB.^90^ To begin forming a structural hypothesis, AlphaFold 3 was employed to model the structures of these G-rich candidate sequences and their plausible aptamer-target complexes. AlphaFold’s internal confidence metrics further supported experimental data with predicted template modeling (pTM) scores above 0.60 for all six aptamer-mCherry structure complexes and yielded aptamer structures displaying compact, G-rich folds, supporting predictions by QGRS mapper. Recent studies indicate updated computational approaches continue to emerge in the literature to inform structural analysis and even screening of aptamers.^91–93^ Further advances in predictive computational tools for apo aptamers and aptamer–target complexes will require sustained investment in structural characterization experiments, the development of standardized analytical methods, and consistent reporting of data in open-access databases.^94–99^

## Conclusion

This work establishes a comprehensive framework for improving the efficiency and fidelity of aptamer discovery through the integration of rationally designed screening libraries target-specific enzymatic partitioning and the CompELS selection platform. Direct binding competition eliminates many concerns of PCR bias and sample handling inherent in multiple rounds of SELEX, and this competition-driven method is applicable to even vastly undersampled libraries. The method is likely even better suited for oversampled libraries in which competition is necessary. In lieu of harsher elution methods such as thermal denaturation to recover bound DNA, the TEV-based enzymatic elution allowed for gentle, yet highly specific recovery of bound complexes while minimizing nonspecific library retention. Comparative parallel selection experiments revealed that CompELS can enable library convergence with fewer enrichment cycles, demonstrating a significant advantage enabled by the competitive binding rounds implemented within CompELS while simultaneously avoiding multiple error-prone PCR amplification steps.

Furthermore, this investigation into the impact of aptamer screening library design provides clear evidence that biasing the initial aptamer screening pool toward a validated, G-rich compositional space is an effective strategy for enriching the pool with functional sequences for this model target. Further validation of these designer libraries, however, requires testing against other protein targets with additional structural features beyond β barrels and compositional elements such as sugar groups. Across both selection platforms, libraries biased toward empirically derived, GT-rich sequence space consistently outperformed screening libraries with random nucleotide distribution or containing prestructured hairpin elements, confirming the success of the Empirically biased screening library. These results agree well with observations of aptamer sequences derived in previous selection experiments by others and strongly suggest that target-binding selection pressure favors a guanine rich region of sequence space such as the compositional design represented in the Empirical screening library. The characterization of enriched sequences further establishes that high guanine content plays a central role in producing functional aptamer sequences possibly due to the increased stacking propensity of guanine nucleobases into tetrad structures. Together, these results are intended to provide a blueprint for testing the adaptability of CompELS to other partitioning strategies and other protein targets. Successful future studies promise an accelerated pathway toward identifying new therapeutic and diagnostic aptamers - either alone or as a hybrid complex called an oligobody - by combining screening library design with a versatile, yet straightforward selection and candidate aptamer recovery platform.

## Methods

### Oligonucleotide and Protein Preparation

All oligonucleotides (Table 4) were synthesized and PAGE purified by Integrated DNA Technologies (IDT, Coraville, IA, USA). Three distinct 90 nucleotide (nt) ssDNA libraries containing a 45 nt central variable central segment with a unique 5 nt barcode downstream from the 5′ end for sample demultiplexing, and two 20 nt fixed base segments. A prior study details sequencing and characterization information for these three libraries.^58^ For PCR enrichment of libraries during screening, universal primers were employed. The forward (5′-GTCAGCATACTCGAGCTCTG-3′) primer is 20 nt and the 20 nt reverse primer (5′-GACAGCGAGTACGAGCATAG-3′) is modified with a double triethylene glycol spacer and a 30 nt long poly dA tail at the 3′ end to facilitate post-PCR separation from amplified template via band excision from denaturing PAGE (Figure S1) detailed in a separate Preparative PCR and ssDNA Generation subsection.

**Table 4:**
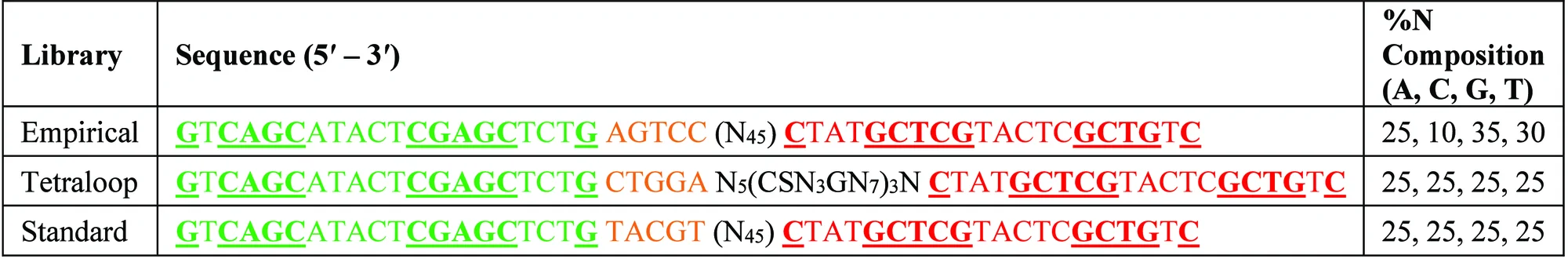
List of Library Nomenclature, Template Primary Structure, and Percentage of Each Base^a^

a Complementary bases (bold, underlined text) between fixed base segments at 5′ (green text) and 3′ (red text) ends are intended to promote imperfect stem formation. Each library template has a barcode (orange text) preceding the central 45-nucleotide long central segment with various design elements included.

In addition to serving as primer binding sites for enzymatic processing, the fixed base segments were designed to create a weak partially hybridized stem (*T*_m_ = 40.8 °C). This partial base-stem serves to preoccupy the fixed base segments and minimize their interaction with the variable central segment. The three libraries were as follows: a nucleotide-biased Empirical library designed with a GT-rich nucleotide bias (35%G 30%T 25%A 10%C) informed by compositional bias observed in 51 DNA aptamers deposited in the Protein Data Bank (PDB), a Tetraloop library containing three embedded CSNNG (where S = G/C and N = A/G/C/T) motifs to promote the self-folding of stable structures and a Standard library control with a standard equimolar nucleotide distribution. Separate lots of template sequences from the vendor were used for initial optimization studies (to minimize both the overall occurrence of nonspecific DNA binding during library-target incubation as well as the recovery of nonspecific DNA during target-DNA complex elution) and then for subsequent screening experiments to minimize cross-contamination between studies. For selection experiments, an equimolar mixture of each library was prepared to create the initial starting pool consisting of approximately 1 × 10^15^ unique molecules. This library mixture served as the starting library for the first round of SELEX. For CompELS, this naive library mixture was split into ten separate aliquots for the ten screening cycles of Stage I CompELS.

The mCherry protein was expressed using a pET Biotin His6 mCherry LIC cloning vector (H6-mCherry), which was a gift from Scott Gradia (Addgene plasmid # 29722; http://n2t.net/addgene:29722; RRID:Addgene_29722). The plasmid was transformed into BL21­(DE3) cells (Thermo Fisher Scientific, Waltham, MA) and a single colony was grown overnight in LB with 50 μg/mL kanamycin. This starter culture was used to inoculate an expression culture which was grown at 37 °C to an OD_600_ of 0.7. Protein expression was then induced with 1 mM IPTG and the culture was incubated for 5 h at 30 °C. Cells were harvested by centrifugation and the His-tagged mCherry was purified from the lysate using HisPur Ni-NTA Resin (Thermo Fisher Scientific, Waltham, MA). Purified mCherry was then site-specifically biotinylated using a Biotin-Protein Ligase (BirA) Kit (GeneCopoeia, Rockville, MD) according to the manufacturer’s protocol. Successful biotinylation was confirmed by a streptavidin-binding native PAGE gel shift assay stained with Coomassie Blue stain (Figure S2 in Supporting Information). After BirA reaction, biotinylated mCherry was then purified with and buffer-exchanged using Pierce 10K MWCO protein concentrators (Thermo Fisher Scientific, Waltham, MA) and stored in 10 mM Tris-HCl pH 8.0.

### SELEX Screening Protocol

For aptamer selection experiments, 30 μL of NeutrAvidin Ultralink resin (Thermo Fisher Scientific, Waltham, MA) resin slurry was added to a Pierce Micro-Spin Column (Thermo Fisher Scientific, Waltham, MA) then blocked twice with 100 μL blocking buffer (20 mM Tris-HCl pH 7.5, 100 mM NaCl, 5 mM KCl, 2 mM MgCl_2_, 0.5 w/v% PEG 8000, 0.1 v/v% Tween-20, 1 w/v% BSA). Then 15 μg of biotinylated mCherry, which included a Tobacco Etch Virus (TEV) protease cleavage site downstream from the biotinylation site, was immobilized on the blocked NeutrAvidin resin. Before library incubation, each library (10^15^ molecules) was suspended in 400 μL aptamer folding (AF) buffer (20 mM Tris-HCl pH 7.5, 100 mM NaCl, 5 mM KCl, 2 mM MgCl_2_, 0.5% w/v PEG 8000, 0.1 v/v% Tween-20) and annealed by heating to 95 °C for 5 min followed by a slow ramp to 25 °C. The folded library was then precleared in a negative screening step by incubation with a bare, mCherry-free resin-packed column. The flow-through library was then incubated with the immobilized mCherry target for 15 min at 25 °C. Unbound sequences were partitioned by centrifugation for 1 min at 10,000 *g*. After partitioning, the column was washed 15 times with 200 μL stringent wash buffer (AF buffer supplemented with 2 M NaCl and pH 8.5). Following stringent washes, the column was then equilibrated twice with 200 μL AF buffer for 5 min. For elution of aptamer candidate-mCherry complexes from the column, 20 μL of TEV cleavage solution (containing 20 units of TEV protease (New England Biolabs, Ipswich, MA), 1x TEV buffer (NEB), 1x AF) was incubated with the column for 30 min at room temperature with constant rotation. The eluate was then collected by transferring the column to a fresh 1.7 mL tube and centrifugation for 2 min at 10,000 *g*.

### Two-Stage CompELS Screening Protocol

For CompELS, all aspects of target immobilization, preblocking resin and column, library folding, preclearance with negative target, positive target incubation including stringent washing and re-equilibration, and TEV protease elution steps (only at the end of Stage I and Stage II CompELS) were kept identical to that of SELEX (detailed above) except for the library partitioning strategy. In CompELS, after annealing and preclearance with negative target, the folded library is first split into 10 equivalent volumes. Each aliquot is then separately incubated with the mCherry-immobilized resin packed in the column with a centrifugation partitioning step between each incubation round to remove unbound DNA. Importantly, there are no intermittent PCR amplification or target-DNA complex dissociation and elution steps that occur between each incubation and binding competition round with a fresh library aliquot during Stage I of CompELS. DNA-mCherry complexes eluted via specific TEV cleavage following Stage I CompELS are then PCR amplified as detailed in the next subsection. This now enriched library is then again split into 10 equivalent volumes and incubated with fresh immobilized mCherry in 10 consecutive rounds of competitive binding and centrifugation partitioning steps to complete Stage II CompELS. After completing Stage II, target-bound DNA is once again isolated via specific TEV cleavage elution of DNA-target complexes and then PCR amplified.

### Quantitative PCR (qPCR) and Melt-Curve Analysis

After TEV cleavage elution of aptamer-target complexes, 2 μL from the eluate was collected and used for qPCR library quantitation and to monitor library convergence. Reactions (20 μL) were prepared using PowerUp SYBR Green PCR Master Mix (Thermo Fisher Scientific, Waltham, MA) and 500 nM of each universal forward and reverse primer. Thermal cycling consisted of 95 °C for 15 s, 60 °C for 30 s and 72 °C for 20 s for 40 cycles. Concentration standards were prepared by series dilution of an equimolar mixture of each library. Melt curve analysis was performed immediately after PCR in +2 °C increments from 60 to 95 °C.

### Preparative PCR and ssDNA Generation

Preparative PCR was performed on the remaining eluate from each SELEX or CompELS enrichment cycle using Platinum II Taq Hot Start DNA Polymerase supplemented with 5x Platinum GC Enhancer and 500 nM of the universal forward primer and the modified universal reverse primer with a poly dA tail at the 5′ end. The number of PCR cycles used for library enrichment was determined from qPCR of the eluate aliquot (detailed in above subsection) and monitoring the maximum number of cycles (up to 20) before the reaction plateaued to minimize PCR amplification errors and undesirable side-products. Thermal cycling consisted of an initial 95 °C for 5 min activation step followed by cycles of denaturation at 95 °C for 15 s and annealing/extension at 65 °C for 15 s. The resulting dsDNA library was then prepared for strand separation by mixing to a final composition of 50% formamide and 1x loading dye followed by heating at 95 °C for 15 min. The sample was then loaded in a 13 w% polyacrylamide gel with 8 M urea and ran at 250 V for 1 h. The ssDNA sense strand (90 nt) was separated from the poly dA-tailed antisense strand (∼120 nt) and visualized by staining with SYBR Gold (Figure S1). The lower sense band (90 nt) was excised and recovered by an overnight crush-and-soak elution method followed by spin column purification (Monarch PCR & DNA Cleanup Kit, New England BioLabs Inc. Ipswich, MA). The purified ssDNA was quantified by A_260_ (NanoDrop) and used for the next enrichment cycle of screening or NGS preparation discussed next.

### Next Generation Sequencing (NGS) and Data Analysis

The naive and enriched library pools were prepared for sequencing using a two-step amplicon protocol previously shown to enable successful sequencing of single amplicon libraries with the Illumina platform.^98^ In PCR1, 10 ng of library was amplified for 8 cycles using NEBNext Q5 Ultra II Master Mix and primers containing Nextera sequences followed by heterogeneous spacers (1–10 random nucleotides) that artificially boost base diversity during sequencing through a frame-shifting effect. The samples were then loaded in a 3% agarose gel and the 157–178 nt product excised then purified with spin column purification. For PCR2, the PCR1 product was amplified again with the Q5 Ultra II Master Mix for 6 cycles to add dual indices and P5/P7 adapters (xGen NXT UDI Primers, IDT). The PCR2 product was then loaded in a 3% agarose gel and the 230–259 nt product was excised then purified using Monarch DNA Cleanup Kit (New England BioLabs, Ipswich, MA). A 13 w% denaturing urea PAGE was used to confirm the product of the correct length after each PCR step. The prepared libraries were then quantified (NEBNext Library Quant Kit, New England Biolabs, Ipswich, MA) and sequenced on an Illumina MiSeq system (v2 Nano kit, 2 × 150 bp) with <2% PhiX spike-in. Raw FASTQ files (DOI: 10.5281/zenodo.19432013) were processed and paired-end reads were assembled using the PEAR tool from the Galaxy toolkit (usegalaxy.org).^89^ The assembled reads were then trimmed of their universal forward and reverse primer sequences using a custom Python script and filtered to retain only sequences of the correct length. These reads were then demultiplexed using a custom Barcode Splitter tool in the Galaxy toolkit based on their unique 5 nt barcodes. The FASTAptamer 2.0 toolkit (https://fastaptamer2.missouri.edu) was used to perform sequence clustering and sequence family identification.^94^ Finally, a custom Python script was used to parse the FASTAptamer output and track frequencies/enrichment of the top sequence clusters across all selection rounds to analyze enrichment dynamics (Figure S3).

### Aptamer Candidate Characterization

Electrophoretic mobility shift assay (EMSA) consisted of each aptamer used at 0.5 nM concentration in AF buffer and folded by annealing for 10 min at 95 °C then cooling to 25 °C at a rate of 0.1 °C/sec. For each binding curve, a serial dilution of mCherry protein was prepared in AF buffer and each dilution mixed with aptamer to create a final incubation volume of 25 μL with mCherry concentration ranging from 10 nM to 8 μM. The reaction was then incubated for 45 min at 25 °C. After the reaction, the samples were prepared for native PAGE by mixing with a loading buffer containing 1x AF and 20% Glycerol (v/v). The samples were then loaded in a 10 well 13% polyacrylamide gel and ran at 180 V for 2.5 h using 0.5x Tris-Borate buffer (pH 8.0) inside a refrigerator at 4 °C. The 10-well gels were prepared 37.5:1 acrylamide: bis-acrylamide in Novex 1.5 mm gel cassettes. Gels were then stained with SYBR Gold and imaged using a Typhoon FLA 9500 (GE, Chicago, IL) with excitation at 495 nm and a pixel size of 100 μm. Gel images were analyzed band intensities quantified with ImageJ.

### *In Silico* Analysis

The potential for G-quadruplex formation was assessed using the QGRS Mapper web server (https://bioinformatics.ramapo.edu/QGRS/analyze.php). The three-dimensional structural modeling of aptamer-mCherry complexes with associated potassium and sodium ions was performed using AlphaFold 3 via the AlphaFold server (https://alphafoldserver.com).

## Supplementary Material



## Data Availability

The FASTQ data are available online (DOI: 10.5281/zenodo.19432013).

## Notes

This material is available free of charge via the Internet at the following link. The Supporting Information is available free of charge at https://pubs.acs.org/doi/10.1021/acsnanomed.6c00140.

Polyacrylamide gel electrophoresis following library purification (Figure S1), polyacrylamide gel electrophoresis to verify mCherry binding to streptavdin (Figure S2), sequence cluster enrichment as a function of enrichment cycle number (Figure S3), and electrophoretic mobility shift assays of aptamer candidate (Figure S4) (PDF)
