# Supplementary material for: CompELS vs SELEX: Comparative Aptamer Discovery Integrating Rational Libraries and Enzymatic Elution
Source: ACS Nano Med. 2026 Sep 2;1(9):2442–55. doi: 10.1021/acsnanomed.6c00140 (PMC13674330; doi:10.1021/acsnanomed.6c00140)
Supplement: Supplementary file 1 [file nm6c00140_si_001.pdf]

Supporting Information

CompELS vs. SELEX: Comparative Aptamer Discovery Integrating Rational Libraries and Enzymatic Elution

Steven Ochoa,[a] and Valeria Tohver Milam*[a, b]

[a] School of Materials Science & Engineering, Georgia Institute of Technology
Atlanta, GA

[b] Parker H. Petit Institute for Bioengineering and Biosciences, Georgia Institute of Technology
Atlanta, GA

E-mail: valeria.milam@mse.gatech.edu

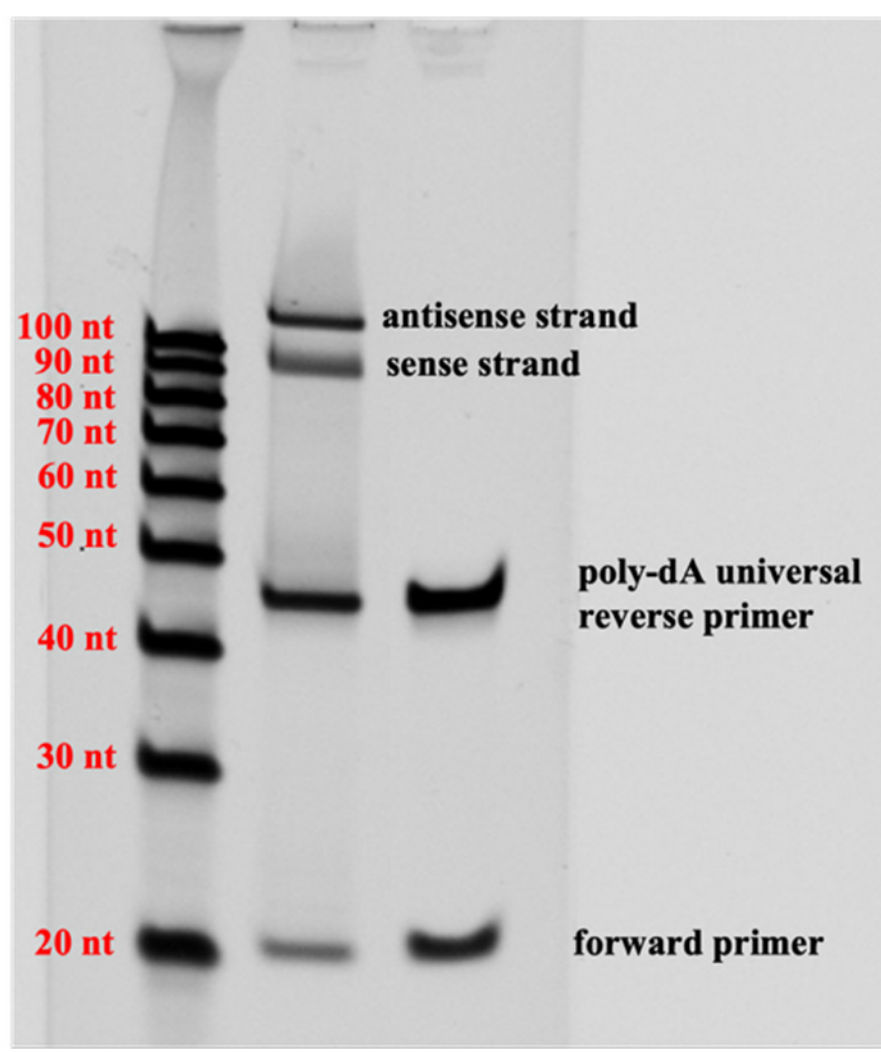

Figure S1. Denaturing PAGE strand separation of amplified structured library used to excise ssDNA sense strand and generate screening library for each enrichment cycle.

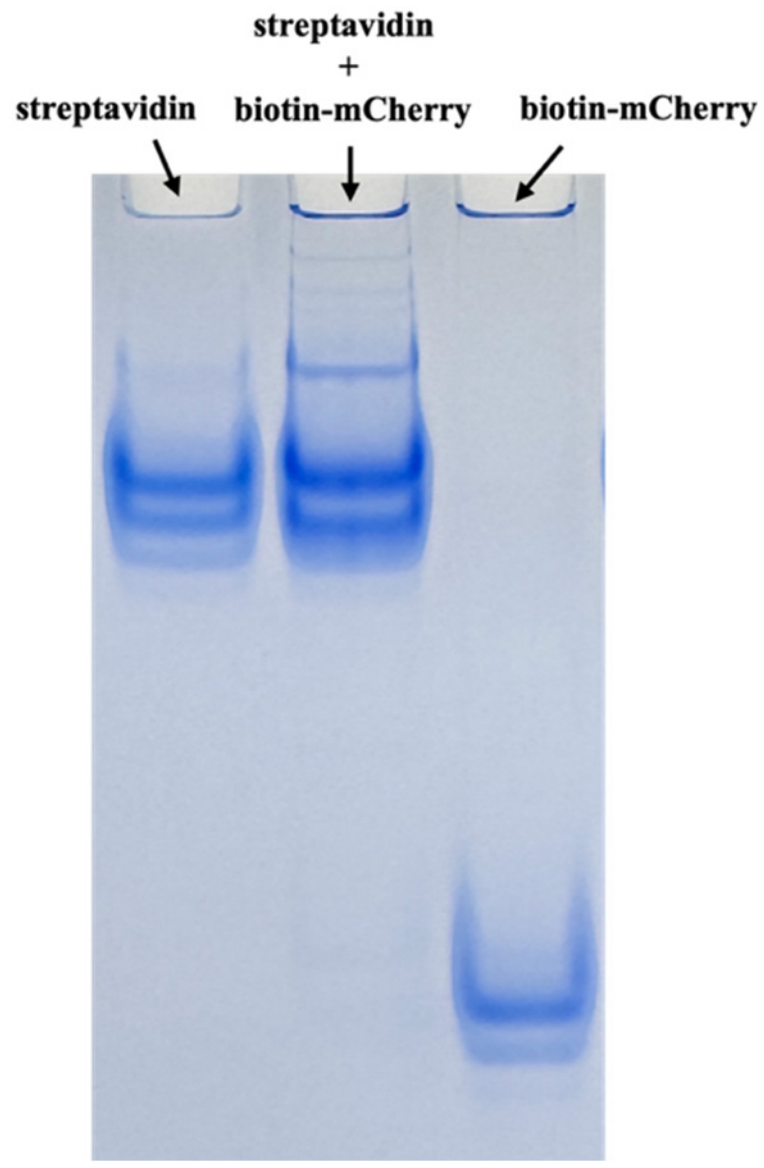

Figure S2. EMSA analysis of biotinylated-mCherry interaction with streptavidin.

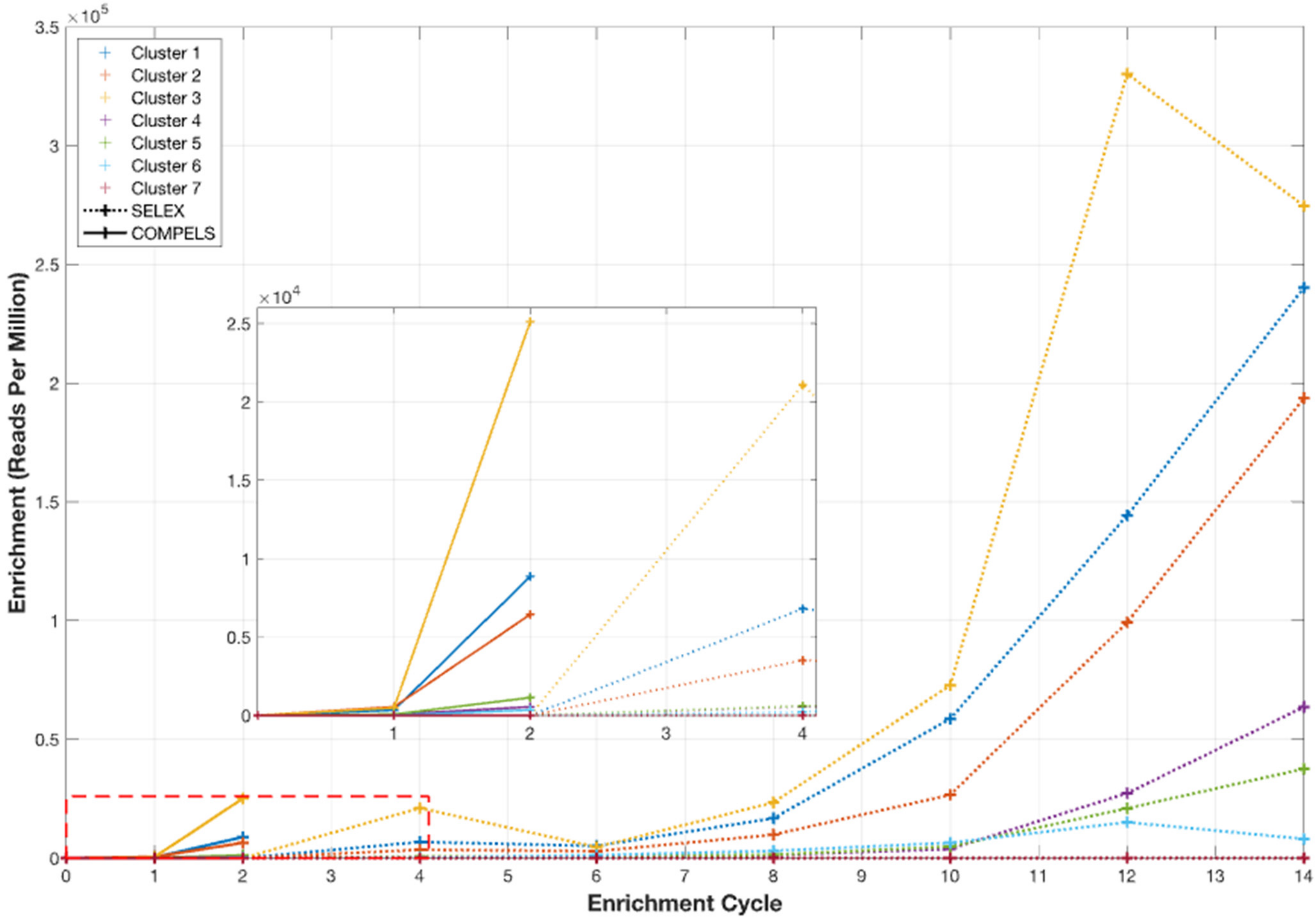

Figure S3. Comparison of sequence cluster enrichment (Reads Per Million, RPM) over 14 SELEX enrichment cycles (dotted lines) and 2 CompELS enrichment cycles (following each of two CompELS stages) (solid lines). The inset magnifies critical early cycles (0-4) to pinpoint aptamer emergence for each selection platform. Sequence clusters generated using an LED = 10.

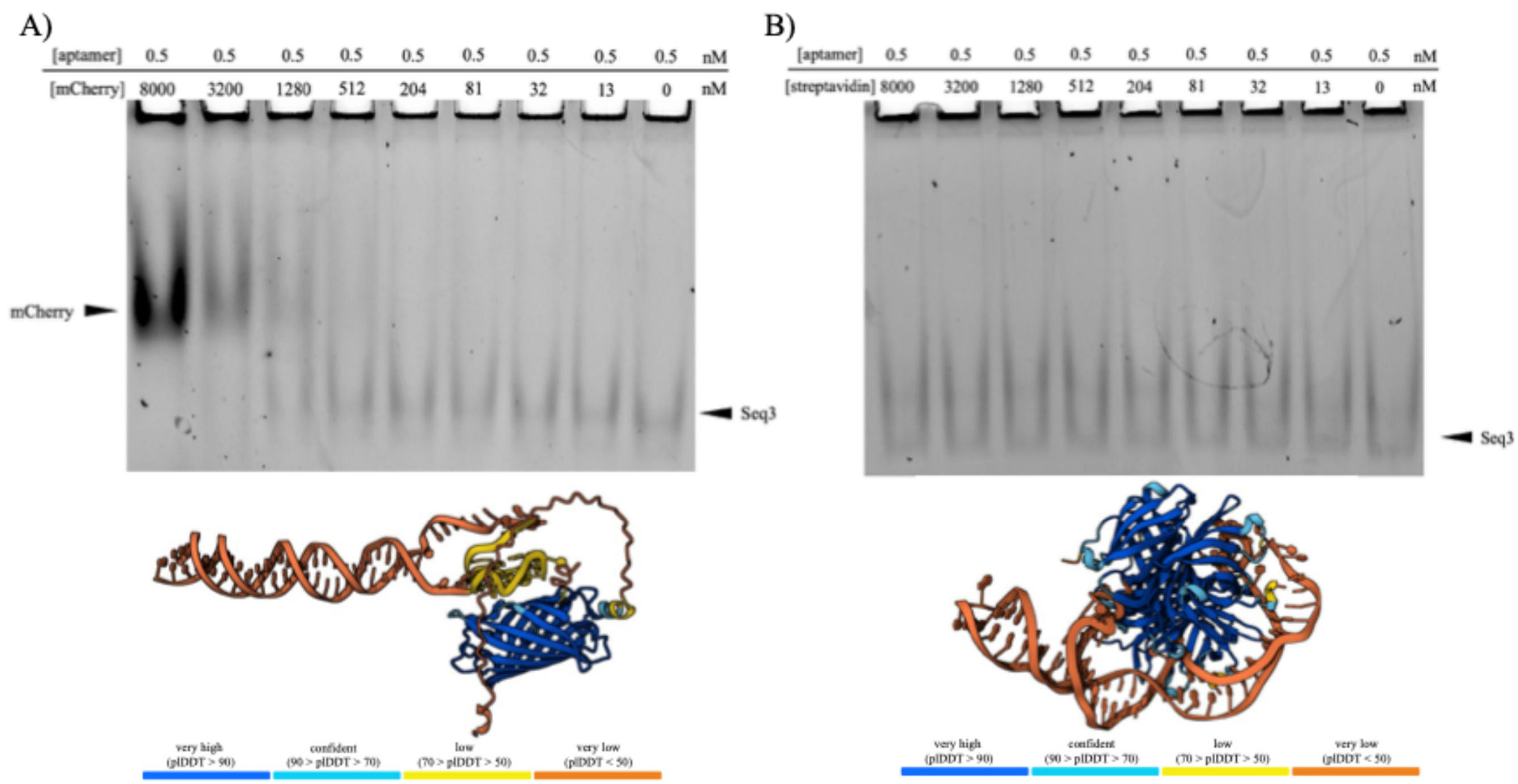

Figure S4. A) Binding validation and structural prediction for aptamer candidate Seq3. (top) Electrophoretic Mobility Shift Assay (EMSA) used to determine the binding affinity of the aptamer to mCherry. (bottom) AlphaFold 3 structural model of the mCherry-aptamer complex. B) Nonspecific binding validation and structural prediction for aptamer candidate Seq3. (top) Electrophoretic Mobility Shift Assay (EMSA) used to determine the binding affinity of the aptamer to streptavidin. (bottom) AlphaFold 3 structural model of the streptavidin-aptamer complex. The model is colored by its pLDDT (predicted Local Distance Difference Test) score, a per-residue estimate of confidence ranging from 0-100. A score >90 (blue) is considered 'very high' confidence, 70-90 (light blue) is 'confident', 50-70 (yellow) is 'low' confidence, and < 50 (orange/red) is 'very low' confidence, which may indicate an unstructured region.
